# Weight recurrence after Sleeve Gastrectomy versus Roux-en-Y gastric bypass: a propensity score matched nationwide analysis

**DOI:** 10.1007/s00464-022-09785-8

**Published:** 2023-02-06

**Authors:** Erman O. Akpinar, Ronald S. L. Liem, Simon W. Nienhuijs, Jan Willem M. Greve, Perla J. Marang-van de Mheen, the Dutch Audit for Treatment of Obesity Research Group, L. M. de Brauw, S. M. M. de Castro, S. L. Damen, A. Demirkiran, M. Dunkelgrün, I. F. Faneyte, J. W. M. Greve, G. van ’t Hof, I. M. C. Janssen, E. H. Jutte, R. A. Klaassen, E. A. G. L. Lagae, B. S. Langenhoff, R. S. L. Liem, A. A. P. M. Luijten, S. W. Nienhuijs, R. Schouten, R. M. Smeenk, D. J. Swank, M. J. Wiezer, W. Vening

**Affiliations:** 1 Department of Surgery, Maastricht University Medical Center, NUTRIM School for Nutrition and Translational Research in Metabolism, Maastricht, The Netherlands; 2 Scientific Bureau, Dutch Institute for Clinical Auditing, Leiden, The Netherlands; 3 Department of Surgery, Groene Hart Hospital, Gouda, The Netherlands; 4 Dutch Obesity Clinic, The Hague & Gouda, The Netherlands; 5 Department of Surgery, Catharina Hospital, Eindhoven, The Netherlands; 6 Department of Surgery, Zuyderland Medical Center, Heerlen, The Netherlands; 7 Dutch Obesity Clinic South, Heerlen, The Netherlands; 8 Department of Biomedical Data Sciences, Medical Decision Making, Leiden University Medical Center, Leiden, The Netherlands

**Keywords:** Weight recurrence, Non-responder, Total weight loss, Bariatric surgery, Sleeve gastrectomy, Roux-en-Y gastric bypass

## Abstract

**Background:**

Literature remains scarce on patients experiencing weight recurrence after initial adequate weight loss following primary bariatric surgery. Therefore, this study compared the extent of weight recurrence between patients who received a Sleeve Gastrectomy (SG) versus Roux-en-Y gastric bypass (RYGB) after adequate weight loss at 1-year follow-up.

**Methods:**

All patients undergoing primary RYGB or SG between 2015 and 2018 were selected from the Dutch Audit for Treatment of Obesity. Inclusion criteria were achieving ≥ 20% total weight loss (TWL) at 1-year and having at least one subsequent follow-up visit. The primary outcome was ≥ 10% weight recurrence (WR) at the last recorded follow-up between 2 and 5 years, after ≥ 20% TWL at 1-year follow-up. Secondary outcomes included remission of comorbidities at last recorded follow-up. A propensity score matched logistic regression analysis was used to estimate the difference between RYGB and SG.

**Results:**

A total of 19.762 patients were included, 14.982 RYGB and 4.780 SG patients. After matching 4.693 patients from each group, patients undergoing SG had a higher likelihood on WR up to 5-year follow-up compared with RYGB [OR 2.07, 95% CI (1.89–2.27), *p* < 0.01] and less often remission of type 2 diabetes [OR 0.69, 95% CI (0.56–0.86), *p* < 0.01], hypertension (HTN) [OR 0.75, 95% CI (0.65–0.87), *p* < 0.01], dyslipidemia [OR 0.44, 95% CI (0.36–0.54), *p* < 0.01], gastroesophageal reflux [OR 0.25 95% CI (0.18–0.34), *p* < 0.01], and obstructive sleep apnea syndrome (OSAS) [OR 0.66, 95% CI (0.54–0.8), *p* < 0.01]. In subgroup analyses, patients who experienced WR after SG but maintained ≥ 20%TWL from starting weight, more often achieved HTN (44.7% vs 29.4%), dyslipidemia (38.3% vs 19.3%), and OSAS (54% vs 20.3%) remission compared with patients not maintaining ≥ 20%TWL. No such differences in comorbidity remission were found within RYGB patients.

**Conclusion:**

Patients undergoing SG are more likely to experience weight recurrence, and less likely to achieve comorbidity remission than patients undergoing RYGB.

Bariatric surgery is effective in achieving sustained weight loss, comorbidity reduction, and improved quality of life for patients with morbid obesity [1–4]. However, some patients will experience weight recurrence after initially achieving adequate weight loss following bariatric surgery [5–7] .

Weight recurrence is known to be associated with poor clinical outcomes such as comorbidity deterioration and worsened quality of life [6, 8–10]. Although the definition of weight recurrence is still up for debate [11], with arbitrary thresholds showing a wide variety of results [12, 13], patients with significant weight recurrence are potential candidates for revision surgery which makes it important to identify such high-risk patients. Weight recurrence is multifactorial and associated with lifestyle, hormonal, genetic, metabolic factors, and the type of bariatric procedure [6, 8]. Literature has shown that around 25% of patients undergoing Roux-en-Y gastric bypass (RYGB) will show inadequate weight loss (non-response) or weight recurrence in the long term [7, 14]. A recent retrospective study showed that patients undergoing Sleeve Gastrectomy (SG) more often have weight recurrence than patients undergoing RYGB [5]. However, this recent study did not adjust for confounding by indication, even though there may be underlying factors why some patients receive SG or RYGB, which makes it prone to bias as it does not enable fair comparison by balancing out the measured confounders on average between treatment groups [15]. In addition, studies that compare the results of weight recurrence between RYGB and SG remain scarce in the literature, in particular among patients initially achieving adequate weight loss. More evidence is imperative for surgeons to consider the risks of weight recurrence depending on the type of primary bariatric procedure, particularly for high-risk patients.

Therefore, this nationwide study will compare patients undergoing primary RYGB or SG on the extent of weight recurrence up to 5 years of follow-up after initial adequate weight loss at 1 year and assess the associated effect on remission of comorbidities.

## Methods

### Study design

This population based study used data from the Dutch Audit for Treatment of Obesity (DATO). The DATO is a mandatory nationwide audit in which all bariatric procedures are registered since 2015. Previous verification of the DATO data has shown the validity of the data [16]. In accordance with the Dutch Institute for Clinical Auditing (DICA) regulations and following the ethical standards as stated in Dutch law, no informed consent from patients was needed as this is an opt-out registry. This study was approved by all the scientific committee members of the DATO (reference number 2022-16).

### Patient selection

Patients who underwent a primary Sleeve Gastrectomy or Roux-en-Y Gastric Bypass between 2015 and 2018 were identified. Inclusion criteria were achieving ≥ 20% Total Weight loss (TWL) at the first year of follow-up and having at least one subsequent follow-up measurement between 2 up to 5 years. Patients undergoing revision surgery during the 2–5 year follow-up were excluded. The time frame to determine weight loss in the DATO consists of the follow-up year with a range of ± 3 months, meaning that patients could have, e.g., their 1-year follow-up visit between 9 and 15 months after the primary surgery.

### Outcome parameters

The primary outcome of this study was ‘weight recurrence (WR)’, defined as ≥ 10% weight increase from Nadir during the last recorded follow-up between 2 and 5 years. Nadir (lowest recorded weight) was determined in the 1st year of follow-up, conditional on achieving ≥ 20% TWL given inclusion criteria. Secondary outcomes included achieving ≥ 20% TWL or ≥ 50% Excess Weight Loss (EWL) at last recorded follow-up, WR without maintaining 20% TWL at last recorded follow-up, and comorbidity remission for hypertension (HTN), gastroesophageal reflux disease (GERD), type 2 diabetes (T2D), dyslipidemia, obstructive sleep apnea syndrome (OSAS), and osteoarthritis at last recorded follow-up.

### Statistical analysis

Baseline characteristics between the two treatment groups were compared using the Chi-square test for categorical variables and depending on the distribution the *t*-test or Mann–Whitney *U* test for continuous variables. To evaluate the association between WR and type of procedure, all variables with a *p*-value < 0.10 in univariable analyses were included in the multivariable logistic regression model to compare RYGB and SG on WR, adjusted for baseline characteristics and year of follow-up. Baseline characteristics were gender, age, body mass index (BMI), American Society of Anesthesiologists (ASA) classification, T2D, HTN, GERD, OSAS, dyslipidemia, and osteoarthritis. In addition, year of follow-up was included because the duration of follow-up is described to be associated with weight recurrence [17]. Multicollinearity was assessed in all models with the Variance Inflation Factor not exceeding 2. Additionally, the two treatments were matched to adjust for confounding by indication as the patient-mix undergoing the two procedures has been shown to be systematically different [18]. Patients were matched 1:1 on all aforementioned characteristics and year of follow-up, using the nearest neighbor method with a caliper of 0.20 [15]. A standardized mean difference < 0.1 was considered to indicate balanced groups. After matching, propensity score matched analysis were conducted to evaluate the association between RYGB and SG on WR, adjusted for the propensity score. Similar analyses were done to compare the secondary outcomes between the matched groups.

Secondary outcomes were further explored within treatment groups among patients experiencing WR. The Chi-square test was utilized to analyze differences within the (un)matched RYGB group by comparing patients who experienced WR without maintaining 20% TWL with patients who maintained 20% TWL from starting weight. The same analysis was done for the SG group. All statistical analyses were performed in R version 3.4.2. A *p*-value < 0.05 was considered statistically significant in all analyses.

## Results

**Fig. 1:**
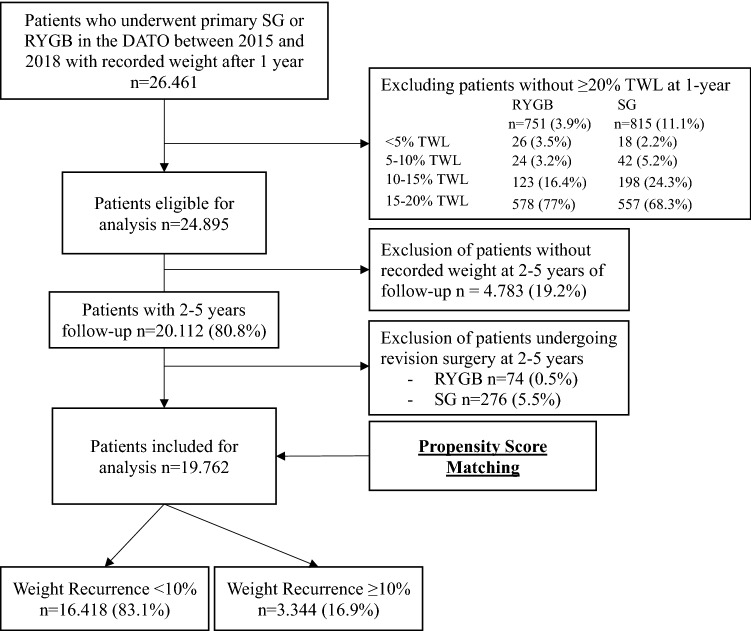
Flowchart of included patients DATO Dutch Audit for Treatment of Obesity, RYGB Roux-en-Y Gastric Bypass, SG Sleeve Gastrectomy and TWL Total Weight Loss

Between 2015 and 2018 a total of 24.895 patients undergoing primary RYGB or SG who achieved ≥ 20%TWL at 1-year follow-up were eligible for analysis. Of these, 19.762 (79.4%) patients were included as they had an additional follow-up measurement between 2–5 years and did not undergo revision surgery, with 4780 patients undergoing primary SG and 14,982 patients undergoing primary RYGB (Fig. 1). The follow-up percentages for the 2nd, 3rd, 4th, and 5th year among eligible patients given their year of operation were 89.3%, 70%, 58%, and 44.6%, respectively. Baseline characteristics between the two treatment groups are shown in Table 1. Patients undergoing SG on average were younger and had a higher BMI. In addition, patients undergoing SG were more often male and had higher ASA classification but less often had T2D, HTN, dyslipidemia, GERD, OSAS and osteoarthritis at baseline than patients undergoing RYGB.

**Table 1:** Patient characteristics of patients undergoing primary RYGB or SG between 2015 and 2018

Characteristics	Before matching		*p* value	SMD	After matching		*p* value	SMD
	RYGB	SG			RYGB	SG		
*n*	14,982	4780			4693	4693		
Sex, No. (%)								
Male	2612 (17.4)	1169 (24.5)	< 0.01	0.17	1115 (23.8)	1122 (23.9)	0.88	< 0.01
Female	12,370 (82.6)	3611 (75.5)			3578 (76.2)	3571 (76.1)		
Age, mean (SD)	45.45 (10.69)	41.96 (12.30)	< 0.01	0.30	42.26 (11.14)	42.11 (12.29)	0.53	0.01
BMI mean (SD)	43.22 (4.89)	45.33 (6.36)	< 0.01	0.37	45.08 (5.61)	45.08 (5.97)	0.96	< 0.01
ASA classification, No. (%)								
I–II	8837 (59.0)	2163 (45.3)	< 0.01	0.28	2168 (46.2)	2149 (45.8)	0.71	0.01
≥ III	6145 (41.0)	2617 (54.7)			2525 (53.8)	2544 (54.2)		
T2D, No. (%)								
Not present	11,762 (78.5)	4087 (85.5)	< 0.01	0.18	4008 (85.4)	4008 (85.4)	1.00	< 0.01
Present	3220 (21.5)	693 (14.5)			685 (14.6)	685 (14.6)		
Hypertension, No. (%)								
Not present	9483 (63.3)	3243 (67.8)	< 0.01	0.10	3110 (66.3)	3175 (67.7)	0.16	0.03
Present	5499 (36.7)	1537 (32.2)			1583 (33.7)	1518 (32.3)		
Dyslipidemia, No. (%)								
Not present	11,696 (78.1)	4002 (83.7)	< 0.01	0.14	3866 (82.4)	3918 (83.5)	0.16	0.03
Present	3286 (21.9)	778 (16.3)			827 (17.6)	775 (16.5)		
GERD, No. (%)								
Not present	12,596 (84.1)	4230 (88.5)	< 0.01	0.13	4169 (88.8)	4147 (88.4)	0.50	0.01
Present	2384 (15.9)	550 (11.5)			524 (11.2)	546 (11.6)		
OSAS, No. (%)								
Not present	12,089 (80.7)	3925 (82.1)	0.03	0.04	3851 (82.1)	3855 (82.1)	0.94	< 0.01
Present	2893 (19.3)	855 (17.9)			842 (17.9)	838 (17.9)		
Osteoarthritis, No. (%)								
Not present	7528 (50.2)	2678 (56.0)	< 0.01	0.12	2594 (55.3)	2622 (55.9)	0.57	0.01
Present	7452 (49.7)	2101 (44.0)			2099 (44.7)	2071 (44.1)		
Weight recurrence, No. (%)								
< 10%	12,687 (84.7)	3731 (78.1)	< 0.01	0.17	4097 (87.3)	3655 (77.9)	< 0.01	0.25
≥ 10%	2295 (15.3)	1049 (21.9)			596 (12.7)	1038 (22.1)		

*RYGB* Roux-en-y Gastric Bypass, *SG* sleeve gastrectomy, *BMI* body mass index, *ASA* American society of anesthesiologists, *T2D* type 2 diabetes mellitus, *GERD* gastroesophageal reflux disease, *OSAS* obstructive sleep apnea syndrome, *SMD* standardized mean differences

### Primary outcome

Adjusted for differences in baseline characteristics, Table 2 shows that patients who underwent SG had a higher likelihood to experience WR compared with patients who underwent RYGB [OR 2.07, 95% CI (1.89–2.27), *p* < 0.01]. Additional factors associated with a higher likelihood on WR were longer follow-up, with the 5th year having the highest likelihood [OR 10.9, 95% CI (9.49–12.51), *p* < 0.01]. On the other hand, older patients and those with a higher BMI at primary surgery were less likely to experience WR [OR 0.99, 95% CI (0.98–0.99), *p* < 0.01] and [OR 0.99, 95% CI (0.98–1.00), *p* < 0.01], respectively.

**Table 2:** Multivariable logistic regression analyses of weight recurrence between 2–5 years of follow-up

Multivariable analyses (*n* = 19.762)	Weight recurrence between 2 up to 5 years of follow-up		
	No. (%)^a^	aOR [95% CI]	*p* value
Type of procedure			
RYGB	14,982 (75.8)	Ref	
SG	4780 (24.2)	2.07 (1.89–2.27)	< 0.01
Sex			
Male	3781 (19.1)	Ref	
Female	15,981 (80.9)	0.92 (0.83–1.02)	0.13
Age	19,762 (100)	0.99 (0.98–0.99)	< 0.01
BMI	19,762 (100)	0.99 (0.98–1.00)	< 0.01
ASA			
I/II	11,000 (55.7)	Ref	
≥ III	8762 (44.3)	0.8 (0.74–0.88)	< 0.01
Hypertension			
Not present	12,726 (64.4)	Ref	
Present	7036 (35.6)	0.93 (0.85–1.02)	0.14
GERD			
Not present	16,826 (85.1)	Ref	
Present	2934 (14.9)	0.97 (0.86–1.1)	0.63
Hyperlipidemia			
Not present	15,698 (79.4)	Ref	
Present	4064 (20.6)	0.97 (0.87–1.08)	0.55
Follow-up (T0 = 1-year)^b^			
2-year (*n* = 19,762)	17,649 (89.3)	Ref	
3-year (*n* = 14,593)	10,225 (70)	3.91 (3.46–4.43)	< 0.01
4-year (*n* = 9482)	5502 (58)	7.59 (6.69–8.6)	< 0.01
5-year (*n* = 4460)	1990 (44.6)	10.9 (9.49–12.51)	< 0.01

Abbreviations: RYGB, Roux-en-y Gastric Bypass; SG, Sleeve Gastrectomy; BMI, body mass index; ASA, American society of anesthesiologists; GERD, gastroesophageal reflux disease; aOR, adjusted odds ratio; CI, confidence interval

^a^The absolute number and percentage are shown for categorical variables and the mean (SD) for continuous variables

^b^Read horizontally; No. and percentage follow-up are calculated based on year of surgery, e.g.: patients with surgery in 2017 could have a recorded follow-up at 3-years, but are not included for year 4 or 5

After matching 4693 patients from both treatment groups, there were no significant differences in baseline characteristics with all standardized differences below 0.1 indicating balanced groups (Table 1). In these matched groups, patients who underwent SG still had a higher likelihood to experience WR compared with RYGB [OR 1.98, 95% CI (1.77–2.21), *p* < 0.01] (Table 3).

**Table 3:** Propensity score matched comparison of SG versus RYGB at 2 up to 5 years follow-up, with RYGB as a reference

	aOR [95% CI]	*p* value
Primary outcome^a^		
≥ 10% weight recurrence	1.98 [1.77–2.21]	< 0.01
Secondary outcome(s)^a^		
≥ 10% WR and < 20% TWL (2 up to 5-years)	1.99 [1.6–2.46]	< 0.01
≥ 20% TWL (2 up to 5-years)	0.36 [0.31–0.42]	< 0.01
≥ 50% EWL (2 up to 5-years)	0.43 [0.38–0.49]	< 0.01
Comorbidity remission^ab^		
T2D		
HbA1c (< 53 mmol HbA1c/mol HbA)	0.69 [0.56–0.86]	< 0.01
Hypertension		
Normotensive (< 120/80 mmHg)	0.75 [0.65–0.87]	< 0.01
Dyslipidemia		
Normal lipid spectrum (LDL, HDL, Triglycerides)	0.44 [0.36–0.54]	< 0.01
GERD		
Absence of symptoms and a normal physiological test (by 24–48 h pH measurement or by gastro-duodenoscopy)	0.25 [0.18–0.34]	< 0.01
OSAS		
No symptoms after preoperative diagnosis of OSAS by means of poly(somno) graphs (PSG), in combination with apnea–hypopnea index (AHI) < 5 and no (more) use of CPAP/BiPAP	0.66 [0.54–0.8]	< 0.01
Osteoarthritis		
No symptoms after pre-operative diagnosis of joint complaints	0.48 [0.41–0.55]	< 0.01

*RYGB* Roux-en-Y Gastric Bypass, *SG* sleeve gastrectomy, *WR* weight recurrence, *TWL* total weight loss, *EWL* excess weight loss, *T2D* type 2 diabetes, *GERD* gastroesophageal reflux disease, *OSAS* obstructive sleep apnea syndrome, *aOR* adjusted odds ratio, *CI* confidence interval

^a^Analysis after matching results in balanced groups and is only adjusted for confounding by indication using the propensity score, thereby comparing patients with the same chance of receiving a procedure

^b^Remission is defined as no medication use in combination with the criteria as stated in the table above

### Secondary outcomes

Within the matched groups, patients who underwent SG were significantly less likely to maintain 20% TWL [OR 0.36, 95% CI (0.31–0.42), *p* < 0.01] or 50% EWL [OR 0.43, 95% CI (0.38–0.49), *p* < 0.01] at their last recorded follow-up compared with RYGB. Furthermore, patients undergoing SG were less likely to achieve comorbidity remission for T2D, HTN, dyslipidemia, GERD, OSAS, and osteoarthritis (Table 3).

Within the matched groups, a total of 596 (12.7%) patients had WR after RYGB and 1038 (22.1%) patients after SG. In addition, patients undergoing SG had a higher likelihood to experience WR without maintaining 20% TWL from starting weight than patients undergoing RYGB [OR 1.99, 95% CI (1.6–2.46), *p* < 0.01]. Matched patients undergoing SG with WR who maintained 20% TWL from starting weight, more often showed comorbidity remission for HTN (44.7% vs 29.4%), dyslipidemia (38.3% vs 19.3%), and OSAS (54% vs 20.3%) than patients who did not maintain 20%TWL after SG (Table 4). Among matched RYGB patients, such a difference in comorbidity remission was not found.

**Table 4:** Concomitant effect of weight recurrence on comorbidity remission between RYGB and SG

Secondary outcomes^a^ at last recorded follow-up	< 20% TWL	≥ 20% TWL	*p*-value
	No. (%)	No. (%)	
a Before propensity score matching			
Unmatched SG patients with WR ≥ 10%	*N* = 461	*N* = 588	
T2D remission	33 (44)	44 (61.1)	0.06
HTN remission	42 (29.2)	80 (44.2)	0.01
Dyslipidemia remission	16 (19.3)	41 (38.3)	0.01
GERD remission	7 (12.7)	9 (11.7)	1.00
OSAS remission	16 (20.3)	54 (52.9)	0.01
Osteoarthritis remission	26 (11.9)	43 (16.8)	0.17

Unmatched RYGB patients with WR ≥ 10%	*N* = 678	*N* = 1617	
T2D remission	92 (51.7)	160 (52.5)	0.94
HTN remission	97 (38.2)	256 (51)	< 0.01
Dyslipidemia remission	61 (43.9)	148 (49.3)	0.34
GERD remission	26 (32.5)	55 (29.7)	0.76
OSAS remission	62 (37.8)	153 (50.2)	0.01
Osteoarthritis remission	53 (16.9)	183 (23.3)	0.03

b. After propensity score matching			
Matched SG patients with WR ≥ 10%	*N* = 459	*N* = 579	
T2D remission	33 (44)	44 (61.1)	0.06
HTN remission	42 (29.4)	80 (44.7)	0.01
Dyslipidemia remission	16 (19.3)	41 (38.3)	0.01
GERD remission	7 (12.7)	9 (11.7)	1.00
OSAS remission	16 (20.3)	54 (54)	< 0.01
Osteoarthritis remission	26 (11.9)	43 (16.9)	0.16

Matched RYGB patients with WR ≥ 10%	*N* = 171	*N* = 425	
T2D remission	16 (55.2)	25 (44.6)	0.49
HTN remission	18 (33.3)	63 (50.8)	0.05
Dyslipidemia remission	10 (38.5)	34 (50)	0.44
GERD remission	8 (50)	5 (18.5)	0.07
OSAS remission	22 (47.8)	39 (56.5)	0.47
Osteoarthritis remission	11 (13.6)	47 (25.4)	0.05

Analysis after matching results in balanced groups, thereby comparing patients with the same chance of receiving a procedure.

*RYGB* Roux-en-Y Gastric Bypass, *SG* sleeve gastrectomy, *WR* weight recurrence, *TWL* total weight loss, *T2D* type 2 diabetes, *HTN* hypertension, *GERD* gastro esophageal reflux disease, *OSAS* obstructive sleep apnea syndrome. Calculations of remission percentages are made for patients in whom the comorbidity was present prior to surgery.

^a^Remission is defined as no medication use in combination with the criteria as stated in the table above.

## Discussion

Knowledge on differences in risks for weight recurrence between bariatric procedures is crucial during pre-operative consultation of patients. The current nationwide study including 19.762 patients, showed that patients who achieved at least 20%TWL at 1-year follow-up after SG had an increased likelihood on weight recurrence, were less likely to maintain 20%TWL and less likely to achieve comorbidity remission at their last follow-up to 5-years compared with similar patients after RYGB. In addition, matched patients with weight recurrence after SG who maintained ≥ 20% TWL more often showed comorbidity remission compared with those who did not maintain 20% TWL.

Weight recurrence has been described to result in lowered quality of life and comorbidity deterioration [19–21]. Factors associated with weight recurrence identified in this study are age, BMI, and longer follow-up, which are in line with current literature [5, 17, 22]. It has to be noted that BMI was not associated with increased weight recurrence, which has been shown to be more likely for patients with a baseline BMI ≥ 50 [23]. The matched patients in this study on average had a BMI of 45, meaning that the difference in weight recurrence between both surgery groups is estimated among patients with mostly BMI < 50. In addition, a previous systematic review showed that patients undergoing SG more often have significant weight recurrence compared with RYGB, although the majority of the included studies had small sample sizes [24]. The current study had much larger sample size due to the nationwide character and used propensity score matching, often referred to as pseudo-randomization, so that it provides stronger evidence for the higher likelihood of patients undergoing SG to experience weight recurrence up to 5-years of follow-up than after RYGB.

Less postoperative weight loss has been described to be associated with higher risks on weight recurrence [17, 25]. Since studies have shown better short-term weight loss results after RYGB than after SG [26], our study included only patients who initially achieved ≥ 20%TWL at 1-year to ensure the same starting point so that we could attribute any difference in outcome to the different procedure rather than the initial difference in weight loss. Despite initially achieving 20%TWL, weight recurrence occurred in 12.7% of patients after RYGB and 22.1% after SG. This suggests that even in patients who initially achieved adequate weight loss, longer follow-up is required to detect weight recurrence in a timely manner. In addition, it suggests that patients may require multiple sequential or parallel treatment strategies such as additional surgery [18] or medical treatment [27] to prevent or treat weight recurrence, as a single bariatric procedure may not always suffice [28–31] .

Comparative studies between RYGB and SG in achieving T2D remission remain controversial. Previous studies have shown that RYGB has better T2D remission than SG at 1 year [32], whereas the difference after 5-years was not significantly different in one study [33], but in favor of RYGB in another study [34]. The latter results are consistent with our finding of a higher likelihood on T2D remission after RYGB among patients with initial adequate weight loss, as well as a lower likelihood on weight recurrence. However, the current study also shows that among patients with weight recurrence there is no difference in T2D remission between patients who maintained the ≥ 20%TWL compared with their starting weight or not, for either treatment groups. A possible explanation could be the initial effect of achieving 20% TWL on T2D remission, as a previous study showed that patients within similar weight change classes show no differences in T2D remission between different procedures [35]. In summary, there is need for larger studies with longer follow-up to confirm the association between weight recurrence and different likelihood of T2D remission between these treatment groups.

The current results support the findings of previous studies showing that RYGB achieves better comorbidity control when compared with patients undergoing SG [36–38]. In addition it suggests that patients undergoing RYGB may be less affected by ≥ 10% weight recurrence and its concomitant effect on comorbidity remission, regardless of maintaining 20% TWL, suggesting more favorable metabolic effects after RYGB compared with SG. Furthermore, these results show that maintaining adequate weight loss after weight recurrence less likely affects comorbidity control. Future studies are needed to investigate when patients will benefit the most of sequential (surgical) treatments when weight recurrence is evaluated in combination with TWL from starting weight and comorbidity control.

There are some limitations that should be noted. First, not all patients completed the 5-year follow-up as this is an ongoing registry, meaning that these estimates may be less precise and that results may be different if all patients have completed the 5-year follow-up. However, since both treatments groups were matched on follow-up in subsequent years, this has not affected the comparison between treatment groups. Second, this study did not include patients who eventually underwent revision surgery, which most likely are patients with the worst outcomes including weight recurrence. In addition, the postoperative complications were not included, which should be taken into account for high-risk patients during shared decision-making. Finally, matching cannot adjust for unmeasured confounders such as surgeon preference, which are assumed to be balanced by matching on the measured confounders. Despite the limitations, this is the first nationwide study on weight recurrence after initially achieving 20%TWL for patients undergoing SG and RYGB. Taking into account the likelihood of weight recurrence, maintaining ≥ 20%TWL, and comorbidity remission, the RYGB could be favored in terms of lower frequency of weight recurrence and more frequent comorbidity remission compared with SG. However, other factors have to be taken into account during shared decision-making for a particular type of procedure, such as complication risks and revision surgery.

## Conclusion

Patients undergoing SG are more likely to experience weight recurrence, and less likely to achieve comorbidity remission than patients undergoing RYGB. In addition, patients with weight recurrence after SG who maintained 20%TWL from starting weight more often showed comorbidity remission than patients not maintaining 20%TWL, suggesting that this should be taken into account when evaluating weight recurrence.

## Abbreviations

*SG*: Sleeve Gastrectomy
*RYGB*: Roux-en-Y gastric bypass
*WR*: weight recurrence
*TWL*: total weight loss
*T2D*: type 2 diabetes
*HTN*: hypertension
*GERD*: gastro-esophageal reflux disease
*OSAS*: obstructive sleep apnea syndrome
*DATO*: Dutch Audit for Treatment of Obesity
*BMI*: body mass index
*ASA*: American Society of Anesthesiologists
